# Supplementary material for: Splice-Junction-Based Mapping of Alternative Isoforms in the Human Proteome
Source: Cell Rep. Author manuscript; Available in PMC 2020 Jan 15. (PMC6961840; doi:10.1016/j.celrep.2019.11.026)
Supplement: 2 [file NIHMS1546469-supplement-2.zip › DF1/PXD010154/Testis/Testis_30_PRPF40A_LNGYSLGGLMVSEMESHPPSQGPGDGER.pdf]

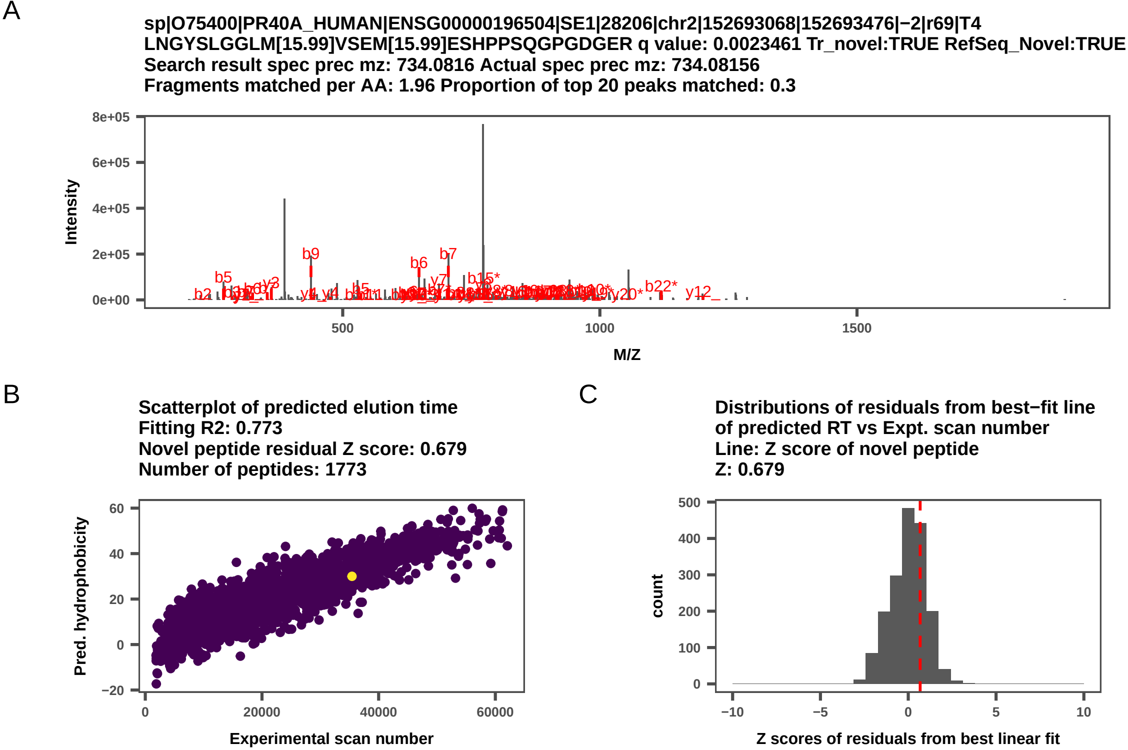
A
sp|O75400|PR40A_HUMAN|ENSG00000196504|SE1|28206|chr2|152693068|152693476|−2|r69|T4
LNGYSLGGLM[15.99]VSEM[15.99]ESHPPSQGPGDGER q value: 0.0023461 Tr_novel:TRUE RefSeq_Novel:TRUE
Search result spec prec mz: 734.0816 Actual spec prec mz: 734.08156
Fragments matched per AA: 1.96 Proportion of top 20 peaks matched: 0.3
Intensity
8e+05
6e+05
4e+05
2e+05
0e+00
b9
b6
b7
b5
b15*
b22*
y12
M/Z
500
1000
1500
B
Scatterplot of predicted elution time
Fitting R2: 0.773
Novel peptide residual Z score: 0.679
Number of peptides: 1773
Pred. hydrophobicity
60
40
20
0
−20
0
20000
40000
60000
Experimental scan number
C
Distributions of residuals from best−fit line
of predicted RT vs Expt. scan number
Line: Z score of novel peptide
Z: 0.679
count
500
400
300
200
100
0
−10
−5
0
5
10
Z scores of residuals from best linear fit
